# Chitosan and Microalgae Nanoparticles: Synergistic Role in Enhancing Drought Stress Tolerance in Wheat Seedlings

**DOI:** 10.3390/plants15050792

**Published:** 2026-03-04

**Authors:** Fatemeh Gholizadeh, Agampodi Gihan S. D. De Silva, Asish Samuel, Zoltán Molnár, Tibor Janda

**Affiliations:** 1 Department of Plant Physiology and Metabolomics, Agricultural Institute, HUN-REN Centre for Agricultural Research, H-2462 Martonvásár, Hungary; 2 Department of Plant Sciences, Széchenyi István University, H-9200 Mosonmagyarovar, Hungary

**Keywords:** chitosan, microalgae biostimulants, drought stress, wheat, seed germination, gene expression

## Abstract

Drought stress is one of the most severe abiotic constraints limiting wheat productivity worldwide, particularly during early developmental stages that determine crop establishment and yield potential. Sustainable, biologically based strategies that enhance drought tolerance without environmental cost are therefore urgently needed. In this study, we evaluated the individual and combined effects of chitosan (Cs), microalgae (Ma) (*Nostoc linckia*, MACC-612), and a chitosan–microalgae nanoparticle formulation (Cs-Ma) on germination performance, early seedling growth, and molecular stress responses in two wheat (Mehregan and MV Nádor) cultivars with contrasting drought sensitivity under polyethylene glycol (PEG)-induced osmotic stress (−2 and −4 MPa). Drought stress significantly reduced germination percentage, germination rate, and radicle and coleoptile development in both cultivars, especially at −4 MPa. Application of Cs and microalgae individually partially alleviated these negative effects; however, the combined Cs-Ma treatment consistently produced the strongest improvements in seedling vigor and biomass accumulation under both moderate and severe drought stress. Evaluation of drought tolerance using tolerance index (TOL), stress tolerance index (STI), and stress intensity (SI) demonstrated that Cs-Ma markedly increased STI and reduced SI across most germination traits, indicating enhanced drought tolerance and lower stress sensitivity, particularly in MV Nádor. These physiological responses were supported by transcriptional reprogramming in radicle tissues, including upregulation of genes involved in polyamine biosynthesis (*TaSPDS*, *TaSAMDC*), phenylpropanoid metabolism (*TaPAL*), and protein protection (*TaHSP70*), along with moderated induction of polyamine catabolism (*TaPXPAO*). Overall, the results reveal a synergistic interaction between chitosan nanoparticles and microalgae biomass, highlighting Cs-Ma as an effective, eco-friendly biostimulant for improving early-stage drought tolerance in wheat.

## 1. Introduction

Wheat (*Triticum aestivum* L.) is one of the most important staple crops worldwide, providing approximately 20% of the global human caloric and protein intake. As a cornerstone of global food security, wheat productivity is increasingly threatened by climate-change-driven environmental stresses, among which drought represents the most pervasive and damaging constraint. Current projections suggest that the proportion of wheat-growing regions exposed to drought stress may increase by nearly 60% by the end of the 21st century, with substantial yield penalties already observed under moderate reductions in water availability [1,2]. Drought stress tolerance indices, such as TOL, STI, and SI, are widely used to quantify plant performance under contrasting water availability and to discriminate between tolerant and sensitive genotypes or treatments. These indices integrate trait responses under both stress and non-stress conditions, providing a robust and comparative framework for evaluating drought tolerance across crops, including wheat, particularly at early developmental stages [3,4]. Consequently, improving drought tolerance in wheat, particularly during early developmental stages that determine stand establishment and yield potential, has become a critical research priority. Drought stress disrupts plant water relations and triggers complex morphological, physiological, biochemical, and molecular responses. At the seedling stage, osmotic stress severely impairs germination, radicle elongation, and biomass accumulation, largely due to altered hormonal balance, oxidative stress, metabolic reprogramming, and reduced protein stability [3]. At the molecular level, drought induces extensive transcriptional reprogramming involving polyamine metabolism, antioxidant defense systems, secondary metabolism, and molecular chaperones, which collectively determine the capacity of plants to tolerate and adapt to water-deficit conditions [5,6]. Among emerging strategies to enhance drought tolerance, environmentally friendly biostimulants have gained increasing attention as sustainable alternatives to conventional agrochemicals.

Chitosan, a natural polysaccharide derived from chitin, has been widely studied due to its biodegradability, biocompatibility, and non-toxic nature. Chitosan functions as a molecular elicitor in plants, activating stress-related signaling pathways mediated by secondary messengers such as hydrogen peroxide, nitric oxide, and calcium ions, thereby enhancing antioxidant defenses, osmotic adjustment, and stress-responsive gene expression [7,8]. Recent advances in nanotechnology have further improved the efficacy of chitosan through nanoparticle formulations, which enhance stability, cellular uptake, and controlled release of bioactive compounds [9,10]. In plant biostimulation, nanoparticle technology typically involves formulating active biomolecules into nanoscale carriers (e.g., chitosan ionotropically crosslinked with tripolyphosphate), which improves dispersion and stability, enables controlled release, and enhances adhesion/uptake at the seed or tissue surface—often translating into stronger physiological and molecular stress-mitigation effects than bulk formulations [11]. Several studies have demonstrated the effectiveness of chitosan nanoparticles in mitigating drought stress in wheat by improving relative water content, photosynthetic performance, antioxidant enzyme activity, and ultimately yield [11,12]. Effects are often accompanied by modulation of stress-related metabolic pathways, including polyamine biosynthesis and redox regulation, which are essential for maintaining cellular homeostasis under water-deficit conditions. In parallel, microalgae have emerged as promising biostimulants due to their rich composition of bioactive compounds, including phytohormones, amino acids, polysaccharides, phenolics, and carotenoids. Application of microalgal biomass has been shown to enhance plant growth, nutrient uptake, photosynthetic efficiency, and tolerance to abiotic stresses, particularly drought [13,14]. Microalgae-derived compounds can influence hormonal signaling, antioxidant capacity, and metabolic balance, thereby improving plant resilience under stress. For example, foliar application of *Chlorella vulgaris* has been reported to significantly improve growth performance, pigment content, and antioxidative defense systems in drought-stressed crops [15].

Despite the demonstrated benefits of chitosan nanoparticles and microalgae when applied individually, their combined use remains largely unexplored. The integration of chitosan nanoparticles with microalgal biomass offers a novel strategy that may exploit the complementary properties of both components, with chitosan acting as a stress signal amplifier and delivery matrix and microalgae providing a diverse pool of bioactive metabolites. Recent studies combining chitosan with biological compounds or extracts have reported synergistic effects on plant growth and stress tolerance, highlighting the potential of such integrated biostimulant systems [16,17]. However, the mechanistic basis of these synergistic effects, particularly at the transcriptional levels, remains unclear, especially in cereal crops such as wheat. Polyamine metabolism plays a central role in drought stress responses, linking nitrogen metabolism, redox homeostasis, and stress signaling. Genes involved in polyamine biosynthesis (*SAMDC*, *SPDS*), catabolism (*PAO*), phenylpropanoid metabolism (*PAL*), and protein protection (*HSP70*) are key molecular hubs in drought adaptation, particularly in root and radicle tissues that directly perceive soil water deficits [5,18,19]. *Nostoc linckia* shows strong prospects as a biofertilizer and biostimulant in crop production, since its nitrogen-fixing capacity and production of growth-promoting compounds can improve soil fertility, enhance plant growth, and support more sustainable reductions in synthetic fertilizer use [20]. Based on these considerations, the objective of this study was to assess whether chitosan, microalgae (*Nostoc linckia*), and their combined nanoparticle formulation can enhance drought tolerance in wheat during germination and early seedling growth. Specifically, the study aimed to (i) evaluate the effects of these biostimulants on germination performance and early growth under PEG-induced osmotic stress, (ii) compare drought responses between two wheat cultivars with contrasting sensitivity, (iii) quantify treatment efficiency using drought tolerance indices (TOL, STI, and SI), and (iv) elucidate underlying molecular mechanisms by analyzing the expression of key genes involved in polyamine metabolism, phenylpropanoid biosynthesis, and protein protection. This study provides mechanistic insight into the synergistic action of chitosan–microalgae nanoparticles as an eco-friendly strategy to enhance early-stage drought resilience in wheat.

## 2. Results and Discussion

### 2.1. Effects of Drought Stress and Treatments on Germination

In this experiment, two wheat cultivars, Mehregan and MV Nádor, were used to evaluate plant responses to drought stress (−2, and −4 MPa) and control conditions, with or without treatment by chitosan (Cs), microalgae strain MACC-612 (Ma) or chitosan–microalgae (Cs-Ma). The analysis of variance revealed that drought stress (Ds), cultivar (C), and treatment (T) significantly affected most germination traits of wheat (Table 1). Drought stress had a significant effect on radicle length (RL), coleoptile length (CL), radicle weight (RW), coleoptile weight (CW), germination percentage (GP), and germination rate (GR), with stronger significance for CL, RW, CW, and GR, indicating the high sensitivity of early seedling growth to decreasing water potential. Cultivar differences were significant (*p* ≤ 0.01) for all measured traits, demonstrating contrasting germination behavior and seedling vigor between Mehregan and MV Nádor under both control and drought stress conditions. Treatments (Ma, Cs, and Cs-Ma) also significantly influenced all traits, with particularly strong effects on biomass-related traits (RW and CW) and coleoptile length (Table 1). The interaction between drought stress and cultivar (Ds × C) was significant for all traits, suggesting that the two cultivars responded differently to drought intensity. Similarly, the interaction between drought stress and treatment (Ds × T) significantly affected seedling growth traits (RL, CL, RW, and CW), while its effect on germination percentage and rate was not significant, indicating that treatments mainly influenced post-germination growth rather than germination initiation under drought. The cultivar × treatment interaction (C × T) was significant for all traits except radicle length, highlighting genotype-dependent responses to microalgae- and chitosan-based treatments. Moreover, the three-way interaction (Ds × C × T) was significant for all traits, confirming that treatment efficiency depended on both cultivar and drought severity (Table 1).

### 2.2. Plant Growth Parameters

Overall, the two wheat cultivars exhibited distinct responses to drought stress and biostimulant treatments during germination and early seedling growth (Table 2). Under control conditions (0 MPa), MV Nádor generally showed higher values for germination-related traits, particularly germination percentage (GP) and germination rate (GR), compared with Mehregan while also responding more strongly to the Ma and combined Cs-Ma treatments. The highest values for radicle length (RL), coleoptile length (CL), radicle weight (RW), coleoptile weight (CW), GP, and GR were consistently observed in MV Nádor under the Cs-Ma treatment at 0 MPa, indicating a strong synergistic effect of the combined treatment in non-stress conditions (Table 2). At moderate drought stress (−2 MPa), germination and seedling growth traits declined in both cultivars; however, MV Nádor generally maintained higher GP, GR, RL, and CL than Mehregan across most treatments. In both cultivars, the Ma and Cs-Ma treatments partially mitigated the negative effects of drought, with the combined Cs-Ma treatment producing the highest RL and biomass-related traits (RW and CW), particularly in MV Nádor. Mehregan showed a stronger reduction in coleoptile length and germination rate under −2 MPa, although improvements were still observed with Ma and Cs-Ma relative to the control. Under severe drought stress (−4 MPa), all germination and seedling traits declined in both cultivars, but MV Nádor consistently maintained higher germination percentage and rate, root and coleoptile length, and seedling biomass than Mehregan across treatments. The combined Cs-Ma application produced the strongest improvement in both cultivars, most notably for root length, coleoptile length, and germination rate indicating superior efficacy under intense water deficit. Mehregan recorded the lowest values at −4 MPa, particularly in the control, although Ma and Cs-Ma provided limited gains, mainly reflected in biomass-related traits (Table 2). Overall, MV Nádor showed greater capacity to sustain early development under moderate and severe drought, especially with Ma or Cs-Ma, whereas Mehregan appeared more sensitive, highlighting cultivar- and trait-dependent responses and the importance of genotype × treatment interactions at early growth stages. The partial recovery of germination and seedling growth following chitosan application aligns with previous findings demonstrating the ability of chitosan to enhance seed vigor and stress tolerance under drought conditions. Chitosan has been shown to act as a stress elicitor, stimulating antioxidant defense systems, improving membrane stability, and facilitating osmotic adjustment during early development [7,8]. Nano-formulated chitosan, in particular, has been reported to improve radicle elongation and seedling biomass in drought-stressed wheat by enhancing bioavailability and cellular uptake, thereby amplifying stress-responsive signaling pathways [10,11]. Likewise, the positive effects of microalgae treatment on germination traits are consistent with earlier studies highlighting the biostimulatory role of microalgal biomass in improving seedling establishment under abiotic stress. Microalgae supply a diverse array of bioactive compounds, including phytohormones, amino acids, and antioxidants, which can promote cell division, enhance nutrient mobilization, and mitigate drought-induced oxidative damage during germination [13,15]. Previous work on wheat shows that microalgae can accelerate germination and enhance early growth by modulating hormonal balance and metabolic activity in young tissues. Notably, the superior performance of the combined chitosan–microalgae (Cs-Ma) treatment supports synergistic biostimulation, consistent with recent studies where chitosan nanoparticles paired with biological extracts or protein hydrolysates outperformed single components by coupling chitosan-mediated stress signaling with metabolite-rich inputs, thereby improving seedling vigor under water-limited conditions [16]. The significant drought × cultivar × treatment interactions further suggest that biostimulant efficacy during germination is genotype-dependent, a pattern widely recognized in wheat drought-tolerance research [3]. The superior performance of Cs-Ma is consistent with the mechanistic premise of nanoparticle delivery—where the chitosan–TPP nanoscale matrix can protect and gradually release bioactive microalgal compounds while facilitating their contact and penetration into young tissues, thereby amplifying downstream stress-response pathways (e.g., antioxidant capacity, osmotic adjustment, and stress-responsive gene regulation) relative to non-nano counterparts. Collectively, these results indicate that combined chitosan–microalgae treatments can mitigate drought-related germination impairment and strengthen early wheat establishment under osmotic stress.

**Figure 1:**
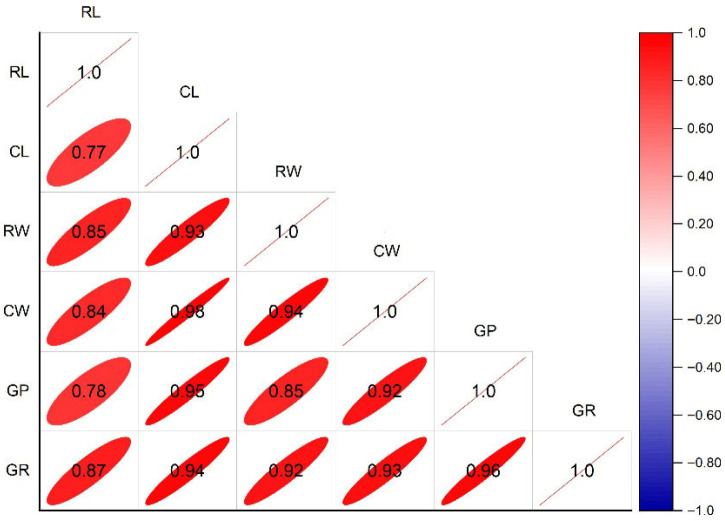
Pearson’s correlation matrix among germination traits of two wheat varieties (Mehregan and MV Nádor) under drought stress (0, −2 and −4 MPa) and across treatments (Ck, Cs, Ma, Cs-Ma). RL, radicle length; CL, coleoptile length; RW, radicle weight; CW, coleoptile weight; GP, germination percentage; and GR, germination rate. Correlations were calculated using aggregated mean values for each cultivar × drought level × treatment combination (N = 24; each mean derived from three biological replicates). Color and ellipse shape indicate the direction and magnitude of correlations.

The correlation analysis revealed strong and positive associations among all germination and early seedling traits. Germination percentage and germination rate exhibited a near-perfect correlation (r = 0.96) (Figure 1). Seedling growth traits, including radicle and coleoptile length and weight, were also highly correlated (r = 0.77–0.94), reflecting coordinated elongation and biomass accumulation during early development. Furthermore, strong correlations between germination traits and seedling vigor parameters suggest that genotypes with superior germination performance tend to produce more vigorous seedlings (Figure 1). Comparable patterns of coordinated trait responses have been consistently documented in wheat exposed to both PEG-induced osmotic stress and field-based drought conditions, highlighting early growth characteristics as robust and informative indicators of drought tolerance [1,4].

**Figure 2:**
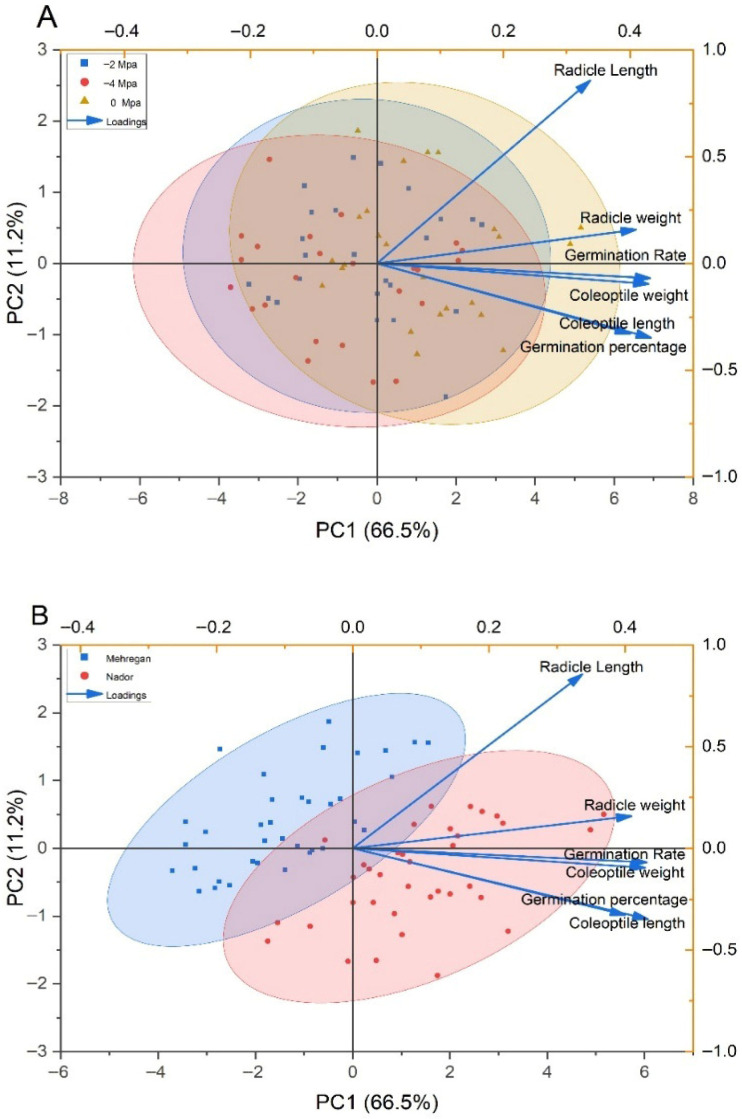
Principal component analysis (PCA) of wheat germination-related traits under drought stress and across varieties. (**A**) Increasing osmotic stress (0, −2, −4 MPa) alters the multivariate trait profile, with separation occurring mainly along PC1 (66.5%) and to a lesser extent PC2 (11.2%). (**B**) Mehregan and MV Nádor exhibit varietal differentiation in the same PCA space. Trait vectors (loadings) indicate that radicle and coleoptile traits and germination metrics contribute predominantly to the positive PC1 direction, with ellipses illustrating group variability.

Principal component analysis explained 77.7% of the total variation, with PC1 (66.5%) associated mainly with germination and seedling vigor traits and PC2 (11.2%) related primarily to radicle development. Control conditions clustered on the positive side of PC1, whereas osmotic stress treatments (−2 and −4 MPa) shifted progressively toward negative PC1 values, indicating reduced germination performance and seedling growth. Radicle length showed the strongest contribution to variation among treatments (Figure 2A). The PCA showed 77.7% of the total variation, with PC1 (66.5%) accounting for most of the variability among samples. PC1 was positively associated with germination percentage, germination rate, radicle length, radicle weight, and coleoptile traits, indicating that these variables jointly contributed to seedling vigor. The MV Nádor group was positioned mainly on the positive side of PC1, reflecting superior germination and early growth performance, whereas Mehregan samples were distributed toward the negative PC1 values (Figure 2B). Similar PCA-based separations between tolerant and sensitive genotypes have been reported in wheat and barley, where PC1 is typically associated with biomass accumulation and elongation traits, and stress treatments cluster along negative PC1 values as water availability decreases [2,3].

### 2.3. Effects of Osmotic Stress on Germination Traits and Stress Indices in Wheat

TOL values indicate that drought stress reduced all germination traits in both cultivars; however, the magnitude of reduction differed by cultivar and treatment. In general, Mehregan exhibited higher TOL values for most traits under control conditions, reflecting higher absolute trait values under non-stress conditions. In both cultivars, the Cs-Ma treatment consistently showed higher TOL values for RL, CL, RW, and CW compared with single treatments, suggesting better maintenance of seedling growth under stress (Table 3).

The stress intensity (SI) values further highlight cultivar differences in drought response. Lower SI values indicate lower sensitivity to drought. Across traits, MV Nádor generally showed lower SI values than Mehregan, particularly for germination percentage and germination rate, suggesting relatively lower stress sensitivity at the germination stage. Among treatments, Cs-Ma and Ma tended to reduce SI compared with the control, indicating mitigation of drought effects (Table 3). The stress tolerance index (STI) clearly discriminated between treatments and cultivars. Higher STI values denote superior performance under both stress and non-stress conditions. For nearly all traits, the Cs-Ma treatment produced the highest STI values, especially in MV Nádor, where marked increases were observed for RL, CL, RW, CW, GP, and GR. This pattern indicates that the combined application of chitosan and microalgae was most effective in enhancing drought tolerance during germination. Mehregan also benefited from Cs-Ma, though STI values were generally lower than those of MV Nádor, reflecting cultivar-dependent responses (Table 3).

Overall, Table 4 demonstrates that drought stress significantly affected germination characteristics in both wheat cultivars, but biostimulant treatments, particularly the combined chitosan–microalgae application, improved drought tolerance, as evidenced by lower SI and higher STI values. Among the two cultivars, MV Nádor showed a more favorable drought tolerance profile at the germination stage, especially when treated with Cs-Ma. Early seedling establishment under drought stress is closely linked to the ability of seeds to sustain cell expansion, reserve mobilization, and biomass allocation under limited water availability. The reductions observed in radicle and coleoptile growth under −4 MPa osmotic stress (Table 3) are consistent with the inhibitory effects of low water potential on cell elongation and metabolic activity during germination [21,22]. Higher STI values under the Cs-Ma treatment, particularly in MV Nádor, indicate an improved capacity to maintain growth and biomass production under stress. STI is widely regarded as an effective index for identifying genotypes and treatments that perform well under both stress and non-stress conditions, reflecting balanced physiological performance rather than stress avoidance alone [23,24].

The lower SI values observed under Cs-Ma suggest mitigation of drought-induced damage, potentially through enhanced osmotic adjustment, membrane stability, or antioxidant activity. Chitosan has been reported to improve stress tolerance by enhancing antioxidant defense systems and regulating water relations, while microalgae-based biostimulants can promote hormonal balance and metabolic efficiency during early growth stages [25,26]. Differences between cultivars highlight inherent genetic variation in stress responsiveness. MV Nádor’s consistently lower SI and higher STI values suggest superior physiological plasticity during germination, which may be linked to more efficient reserve utilization or root system development under osmotic stress traits that are critical for early drought adaptation [22]. Plant–nanoparticle interactions are bidirectional: nanoparticles can alter plant growth and stress physiology (via uptake, signaling and redox effects), while plants can simultaneously modify the nanoparticles’ behavior in the rhizosphere through root exudates that change aggregation, surface chemistry, and dissolution. Reviews emphasize that plant responses can range from beneficial to harmful depending on nanoparticle properties and exposure conditions [27]. Chitosan nanoparticles carrying microalgae could realistically be used in wheat fields, and reports on microalgae-based amendments showing improvements in soil biological activity and wheat growth-/yield-related traits support the idea that microalgae biomass and bioactive compounds can act as both a fertility input and a biostimulant under agronomic conditions [28]. The present study used a PEG-induced osmotic stress assay under controlled conditions to isolate early-stage drought responses during the germination stage. While this approach is valuable for mechanistic screening, it does not fully reproduce the complexity of field drought, where soil physical structure, nutrient dynamics, microbial interactions, fluctuating temperature and evaporative demand, and heterogeneous water distribution strongly influence plant performance. In addition, nanoparticle behavior in soil (e.g., aggregation, adsorption to soil colloids, transport, and biodegradation) and application logistics (dose, timing, and delivery route) can alter bioavailability and efficacy relative to petri-dish exposure. Therefore, the observed benefits of Cs-Ma should be interpreted as evidence of biostimulant potential at the seedling establishment stage, and translation to agronomic outcomes requires validation under greenhouse and field conditions. Future studies should evaluate practical deployment strategies such as seed coating/priming or early-stage soil/foliar applications, optimize concentrations for efficacy and safety, and quantify persistence and environmental fate in soil–plant systems, alongside yield-relevant endpoints under natural drought scenarios.

### 2.4. Gene Expression Responses to Drought Stress

**Figure 3:**
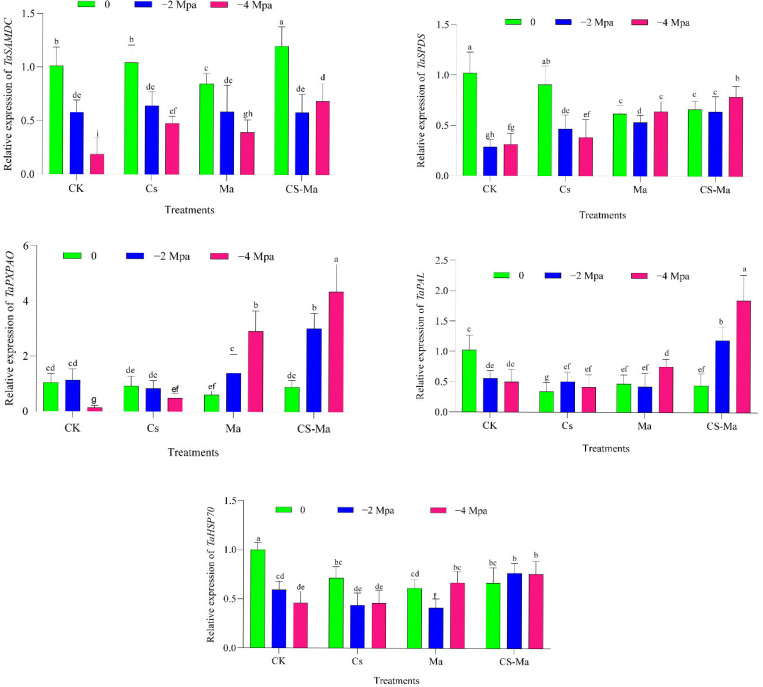
The effect of drought stress (−2, −4 MPa and distilled water as a control) on the expression patterns of spermidine synthase (*TaSPDS*), s-adenosylmethionine decarboxylase (*TaSAMDC*), peroxisomal polyamine oxidase (*TaPXPAO*), phenylalanine ammonia-lyase (*TaPAL*), and heat shock protein 70 (*TaHSP70*) genes in radicle tissue under different treatments (Ck, Cs, Ma, and Cs-Ma) in Mehregan variety. Each column is the mean expression of three technical and biological replicates. Error bars are standard deviations of biological replicates. Lower-case letters above bars indicate mean comparisons from LSD test at *p* < 0.05.

**Figure 4:**
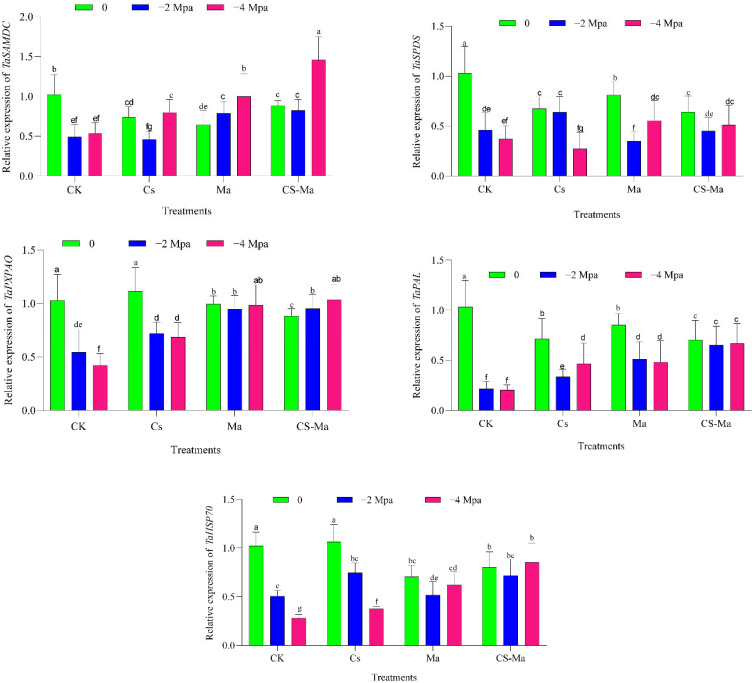
The effect of drought stress (−2, −4 MPa and distilled water as a control) on the expression patterns of spermidine synthase (*TaSPDS*), s-adenosylmethionine decarboxylase (*TaSAMDC*), peroxisomal polyamine oxidase (*TaPXPAO*), phenylalanine ammonia-lyase (*TaPAL*), and heat shock protein 70 (*TaHSP70*) genes in radicle tissue under different treatments (Ck, Cs, Ma, and Cs-Ma) in MV Nádor variety. Each column is the mean expression of three technical and biological replicates. Error bars are standard deviations of biological replicates. Lower-case letters above bars indicate mean comparisons from LSD test at *p* < 0.05.

Drought stress markedly altered the transcriptional regulation of polyamine biosynthesis genes in wheat radicles, with the magnitude and direction of responses depending on both stress intensity and treatment. In the absence of biostimulant application, increasing osmotic stress led to a progressive reduction in *TaSAMDC* and *TaSPDS* expression in both cultivars, particularly under severe stress (−4 MPa), indicating a drought-induced suppression of spermidine and spermine biosynthesis (Figure 3 and Figure 4). This decline is consistent with previous reports showing that drought restricts polyamine biosynthesis due to metabolic limitations and impaired nitrogen assimilation during early seedling development [5,18]. Application of microalgae (Ma) and chitosan–microalgae nanoparticles (Cs-Ma) substantially modified this response. Under both −2 and −4 MPa, these treatments significantly enhanced *TaSAMDC* and *TaSPDS* transcript abundance relative to the control, with the combined Cs-Ma treatment consistently inducing the highest expression levels (Figure 3 and Figure 4). This effect was particularly pronounced under severe drought, suggesting that Cs-Ma treatment effectively preserves polyamine biosynthetic capacity under osmotic stress. Maintenance of polyamine biosynthesis is a key adaptive mechanism, as spermidine and spermine contribute to membrane stabilization, regulation of ion transport, and stress signal transduction [29,30]. The observed upregulation therefore provides a molecular explanation for the improved radicle growth and biomass accumulation in treated seedlings. In contrast to the biosynthetic genes, *TaPXPAO*, encoding a polyamine oxidase involved in polyamine back-conversion and hydrogen peroxide production, exhibited a stress-inducible expression pattern. In control plants, drought stress strongly increased *TaPXPAO* expression, particularly at −4 MPa (Figure 3 and Figure 4), indicating intensified polyamine turnover and oxidative signaling under severe osmotic stress. While moderate activation of polyamine oxidases contributes to stress signaling, excessive activity can lead to overproduction of reactive oxygen species (ROS) and cellular damage [31].

Notably, Ma and Cs-Ma treatments moderated this response. Although *TaPXPAO* expression remained higher under drought than under control conditions, its induction was less extreme in treated plants, especially under Cs-Ma treatment (Figure 3 and Figure 4). This intermediate expression pattern suggests a more balanced regulation of polyamine catabolism, allowing for ROS-mediated signaling without triggering excessive oxidative stress. Such controlled modulation of polyamine turnover has been associated with improved drought tolerance and redox homeostasis in wheat [32,33]. The phenylpropanoid pathway was strongly activated by drought stress, as reflected by increased *TaPAL* expression across both cultivars. In control plants, drought induced *TaPAL* transcription, indicating stimulation of secondary metabolism as part of the stress response. However, this induction was significantly amplified by Ma and Cs-Ma treatments, with the highest *TaPAL* expression consistently recorded under Cs-Ma treatment at −4 MPa (Figure 3 and Figure 4). Phenylalanine ammonia-lyase (PAL) catalyzes the entry step into phenylpropanoid biosynthesis, leading to the production of lignin, flavonoids, and phenolic antioxidants. Enhanced *TaPAL* expression therefore suggests reinforced cell wall structure, improved antioxidant capacity, and increased protection against drought-induced oxidative damage [6,34]. The strong stimulation of *TaPAL* under Cs-Ma treatment provides molecular evidence that the combined formulation enhances structural and biochemical defenses in radicle tissues, contributing to improved drought resilience during early growth.

Drought stress also significantly affected the expression of *TaHSP70*, a key molecular chaperone involved in protein folding and stabilization. In control plants, severe drought stress resulted in reduced *TaHSP70* expression, particularly in radicles exposed to −4 MPa, suggesting impaired protein protection under prolonged osmotic stress (Figure 3 and Figure 4). Such suppression may exacerbate protein misfolding and metabolic dysfunction during early seedling establishment. In contrast, Ma and Cs-Ma treatments maintained or enhanced *TaHSP70* expression under both moderate and severe drought conditions. The highest transcript levels were consistently observed under Cs-Ma treatment (Figure 3 and Figure 4), indicating a strong activation of protein quality control mechanisms. Heat shock proteins, especially *HSP70*, are central to protecting cellular proteins from denaturation, maintaining enzymatic activity, and facilitating recovery following stress release [19,35]. The sustained induction of *TaHSP70* therefore represents a crucial component of the enhanced drought tolerance observed in treated seedlings. Although both wheat cultivars responded positively to biostimulant treatments, clear differences in transcriptional strategy were evident. Mehregan exhibited stronger drought-induced suppression of protective genes under control conditions, followed by pronounced induction under Ma and Cs-Ma treatments, resulting in larger expression amplitudes (Figure 3). In contrast, MV Nádor maintained more stable basal expression across stress levels, with moderate but consistent enhancement under Cs-Ma treatment (Figure 4). These contrasting patterns suggest that Mehregan relies more heavily on inducible stress-responsive mechanisms, whereas MV Nádor exhibits greater transcriptional stability under drought. Such genotype-dependent molecular strategies have been widely reported and emphasize the importance of tailoring biostimulant approaches to cultivar-specific stress responses [3]. Taken together, the gene expression data demonstrate that drought stress disrupts polyamine metabolism, redox balance, secondary metabolism, and protein stability in wheat radicles. The combined chitosan–microalgae treatment consistently mitigated these disruptions more effectively than single treatments by sustaining polyamine biosynthesis (*TaSAMDC*, *TaSPDS*), preventing excessive polyamine catabolism (*TaPXPAO*), enhancing phenylpropanoid-derived antioxidant defenses (*TaPAL*), and reinforcing protein protection mechanisms (*TaHSP70*) (Figure 3 and Figure 4). This coordinated transcriptional reprogramming provides a mechanistic basis for the superior physiological performance observed under Cs-Ma treatment and highlights the synergistic interaction between chitosan-induced stress signaling and microalgae-derived bioactive compounds. Such integration of metabolic, redox, and protein-protection pathways at the molecular level underpins enhanced drought tolerance during early wheat establishment.

## 3. Materials and Methods

### 3.1. Plant Material and Growth Conditions

In the first set of experiments, to investigate the plant responses to drought stress, two wheat varieties (*Triticum aestivum* L.), namely, Mehregan and MV Nádor, were selected, which were obtained from the Agricultural Research Center of Khorasan Razavi Province, Iran and the from the Agricultural Institute, HUN-REN Centre for Agricultural Research Hungary, Martonvásár, Hungary, respectively. These cultivars are among the cultivars of very good quality for bakery. Seeds of uniform size were sterilized by treatment with a solution of 1% sodium hypochlorite for 5 min and then washed extensively with sterile distilled water. In this study, 50 seeds from each variety were put in each petri dish with three repetitions. Filter paper was placed in each petri dish with a diameter of 9 cm, and equal volumes of 4 mL of distilled water as a control and 4 mL from all treatments were added to soak the paper. Seeds soaked with PEG 6000 were placed in a germination chamber at 25 °C for 6 days. The factors included four treatments, i.e., a control (Ck), chitosan (Cs), microalgae strain MACC-612 (Ma) and chitosan–microalgae (Cs-Ma), and three levels of PEG 6000 osmotic potential (−2, −4 MPa and distilled water as a control). Various osmotic potentials were prepared based on the method in [36] to dissolve the required amount of PEG in distilled water at 25 °C. A total of 6 days after the treatments, samples were harvested for measurements of physiological traits, such as coleoptile and radicle weight, coleoptile and radicle length of seed, germination percentage (GP) and seed germination rate (GR). All fresh radicles were immediately frozen in liquid nitrogen and stored at −80 °C for further test. After final germination, GP and GR were calculated by the following equation [33].

$$Germination\;percentage\;(GP)=\left(\frac{Number\;of\;Total\;Germinated\;Seeds}{Total\;Number\;of\;Seeds}\right)\times 100$$

$$Germination\;Rate\;(GR)=\left(\frac{Number\;of\;Germinated\;Seeds}{Day\;of\;First\;Count}+\cdot \cdot \cdot \cdot \cdot \cdot \cdot \cdot \cdot \cdot \cdot \cdot +\frac{Number\;of\;Germinated\;Seeds}{Day\;of\;Final\;Count}\right)$$

### 3.2. Preparation of Microalgae Biomass

**Figure 5:**
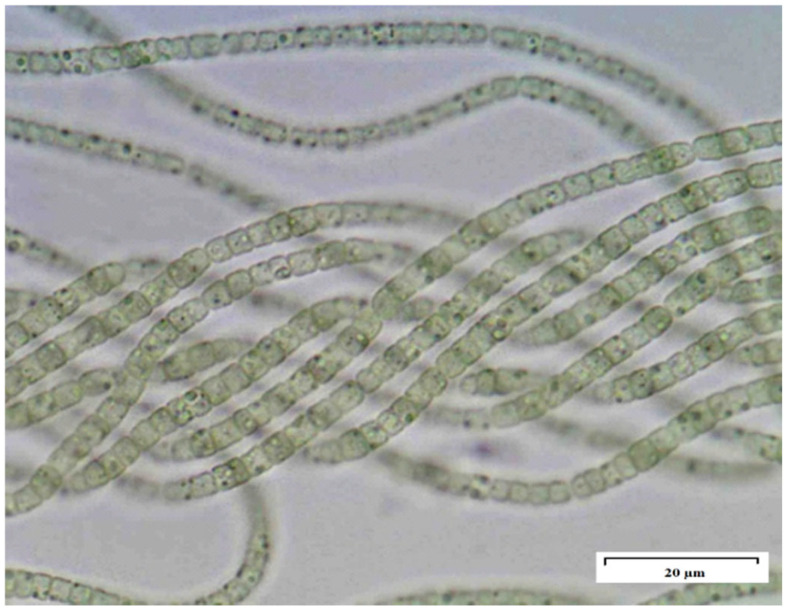
The strain involved in the initial germination test (*Nostoc linckia*, MACC-612) with microalgae biomass. The bar in the figure indicates 20 µm.

The microalgae strain (*Nostoc linckia*, MACC-612), was derived from Mosonmagyaróvár Algae Culture Collection (MACC), Hungary (Figure 5). The strain was incubated at 25 ± 2 °C in a 12:12 light/dark cycle. The microalgae biomass was produced in laboratory culture units. It was illuminated from below with a light intensity of 130 µmol m^−2^ s^−1^ and grown in Tamiya nutrient solution [37], with a starting concentration of 10 mg L^−1^ dry algal weight (dwt). In total, 20 L h^−1^ of filtered compressed air enriched with 1.5% CO_2_ during the light period was used for aerating the culture strain [38]. The cultures grown in these conditions for 10 days were then centrifuged for 15 min at 3000 rpm (Sigma 6 K15, Sigma Laborzentrifugen GmbH, Osterode am Harz, Germany), freeze-dried using Gamma 1–20 (Martin Christ Gefriertrocknungsanlagen GmbH, Osterode am Harz, Germany) and stored at −18 °C. Biomass samples were re-suspended in distilled water and sonicated (VirTis, VirSonic 600 Ultrasonic Cell Disruptor-SP VirTis, Gardiner, MT, USA) 3 min just before plant treatments [39].

### 3.3. Estimation of Drought Tolerant Indices (TOL, STI and SI) in Wheat

Physiological responses to osmotic stress were assessed using tolerance index (TOL), stress tolerance index (STI), and stress intensity (SI), calculated based on trait values under control and drought stress at −4 MPa. These indices were used to quantify stress-induced reductions in growth and biomass partitioning and to compare the relative stress resilience of cultivars and treatments. Drought tolerance indices were calculated using the following formulas [23,24,40]:

$$STI=\frac{YP\;\times \;YS\;}{{\left({\bar{Y}}_{P}\right)}^{2}}$$

*TOL* = *Y_P_* − *Y_S_*

$$SI=1-\frac{{\bar{Y}}_{S}}{{\bar{Y}}_{P}}$$

where the following symbols are used:

*Y_p_* = mean trait value under non-stress (control) conditions.

*Y_s_* = mean trait value under stress conditions (−4 MPa).

${\bar{Y}}_{P}$ = mean of Yp across all cultivar × treatment combinations.

${\bar{Y}}_{S}$ = mean of Ys across all cultivar × treatment combinations.

### 3.4. Preparation of Chitosan and Chitosan–Microalgae Nanoparticles (Cs, Cs-Ma NPs)

Chitosan (Cs) with 75-85% deacetylation (310–375 kDa molecular weight, CAS Number: 9012-76-4) and tripolyphosphate (TPP, CAS Number: 7758-29-4) were obtained from Sigma-Aldrich Co. (St. Louis, MO, USA). Deionized water (DIW) was used for this experiment. Cs-NPs were prepared according to a published method [41]. In summary, a Cs solution was obtained by adding 1 g of Cs powder to 1 L of distilled water and 1000 μL of acetic acid under stirring for 1 h at room temperature. Separately, 0.1 g of microalgae was added to 100 mL of DIW and dissolved by shaking vigorously. The microalgae solution was added to the Cs solution separately. The ratio of Cs to TPP by weight was 2.5:1, so 0.4 g of TPP was dissolved in 50 mL of DIW and slowly added to the Cs-Ma solution.

### 3.5. RNA Isolation and cDNA Synthesis

Total RNA was isolated from radicles of three independent wheat plants per treatment using TRIzol reagent (Invitrogen, Carlsbad, CA, USA) following the manufacturer’s protocol. RNA quantity and quality were assessed using a NanoDrop 2000c spectrophotometer (Thermo Scientific, Waltham, MA, USA). Samples were treated with DNase I and purified using a Direct-zol RNA MiniPrep Kit (Zymo Research, Irvine, CA, USA) according to the manufacturer’s instructions, and cDNA was synthesized from total RNA using M-MLV reverse transcriptase (Promega Corporation, Madison, WI, USA).

### 3.6. Quantitative RT-qPCR

RT-qPCR reactions were performed using PCRBIOSyGreen Mix (PCR Biosystems, London, UK) on a CFX96 Real-Time PCR Detection System (Bio-Rad, Hercules, CA, USA). *TaActin*, which showed stable expression across all conditions, served as the reference gene. Each treatment included three biological replicates and three technical replicates per biological sample. Relative gene expression was calculated using the 2^−ΔΔCT^ formula [42]. Primer pairs for RT-qPCR analysis of key genes are listed in (Table 4). Sequences were adapted from previous studies [18,30,43,44,45].

### 3.7. Statistical Analyses

Treatments were arranged in factorial experiments in a randomized complete block design with three replications. All data were analyzed by two-way analysis of variance (ANOVA) [46]. The statistical analysis of data was performed based on the subsequent LSD range test at a significance level of *p* ≤ 0.05 with four replicates using the SAS software version 9.4 [47]. Correlation analyses and principal component analyses (PCA) were assessed to determine the relationships between the traits using the origin version 2024 software. Correlation coefficients were computed from pooled mean values for each cultivar × drought level × treatment combination (N = 24), with each mean calculated from three biological replicates. GraphPad Prism (version 9.0.1) was employed to create visual representations of the data associated with gene expression patterns.

## 4. Conclusions

Drought stress markedly impaired wheat germination and early seedling growth, particularly under severe osmotic conditions. Although chitosan nanoparticles and microalgae biomass applied individually partially alleviated drought-induced damage, their combined formulation (Cs-Ma) consistently provided the greatest improvement in germination performance and seedling biomass in both cultivars. Evaluation using drought tolerance and sensitivity indices confirmed this effect, as Cs-Ma increased the stress tolerance index (STI) and reduced stress intensity (SI) across most germination traits, indicating enhanced tolerance and lower drought sensitivity. Tolerance index (TOL) values further demonstrated improved maintenance of radicle and coleoptile growth under severe stress, particularly in MV Nádor. These physiological benefits were supported by coordinated transcriptional responses, including upregulation of genes involved in polyamine biosynthesis *TaSPDS* and *TaSAMDC*, phenylpropanoid metabolism *TaPAL*, and protein protection *TaHSP70*, along with moderated induction of polyamine catabolism *TaPXPAO*. Collectively, the results demonstrate a synergistic effect of chitosan–microalgae nanoparticles and support their potential as an environmentally sustainable biostimulant strategy to enhance drought tolerance in wheat plants.

## Figures and Tables

**Table 1:** Analysis of variance for the impact of drought stress (−2, −4 MPa and distilled water as a control) on germination characteristics of two wheat cultivars (Mehregan and MV Nádor).

	Mean Squares						
SOV	DF	RL	CL	RW	CW	GP	GR
R	2	-	-	-	-	-	-
Ds	2	*	**	**	**	*	**
C	1	**	**	**	**	**	**
T	3	*	**	**	**	*	**
Ds × C	2	**	**	**	*	**	*
Ds × T	6	**	**	**	**	ns	ns
C × T	3	ns	*	**	**	**	**
Ds × C × T	6	*	**	**	*	**	*
Error	46	-	-	-	-	-	-

SOV = sources of variations; R = replicate; Ds= drought stress; C = cultivar; T = treatments; DF = degree of freedom; RL = radicle length, CL = coleoptile length, RW = radicle weight, CW = coleoptile weight, GP = germination percentage, GR = germination rate; ns: not significant. *, **: significant at 5% and 1% probability level, respectively.

**Table 2:** The impact of drought stress (−2, −4 MPa and distilled water as a control) on germination characteristics of two wheat cultivars (Mehregan and MV Nádor).

Drought	Cultivars	Treatments	RL (cm)	CL (cm)	RW (g)	CW (g)	GP (%)	GR
0	MV Nádor	Ck	3.70 ^fgh^	3.70 ^bc^	0.025 ^def^	0.029 ^cde^	98.66 ^ab^	25.20 ^b^
		Cs	3.72 ^fgh^	3.07 ^cd^	0.027 ^de^	0.029 ^cde^	98.66 ^ab^	25.10 ^b^
		Ma	4.37 ^cde^	3.74 ^bc^	0.046 ^b^	0.040 ^ab^	98.66 ^ab^	26.50 ^b^
		Cs-Ma	5.39 ^a^	4.57 ^a^	0.053 ^a^	0.042 ^a^	100 ^a^	29.16 ^a^
	Mehregan	Ck	4.49 ^cd^	3.11 ^cd^	0.025 ^def^	0.031 ^cd^	97.33 ^ab^	22.10 ^c^
		Cs	3.65 ^gh^	3.14 ^bcd^	0.025 ^def^	0.030 ^cd^	96.00 ^abc^	22.33 ^c^
		Ma	4.03 ^efg^	3.53 ^bc^	0.034 ^cd^	0.035 ^bc^	97.33 ^ab^	24.83 ^b^
		Cs-Ma	4.59^bc^	3.71 ^bc^	0.039 ^bcd^	0.036 ^bc^	98.66 ^ab^	25.30 ^b^
−2 MPa	MV Nádor	Ck	3.57 ^ghi^	2.69 ^def^	0.023 ^def^	0.022 ^fg^	94.66 ^cd^	19.50 ^cde^
		Cs	3.80 ^fg^	3.20 ^bcd^	0.024 ^def^	0.024 ^efg^	92.00 ^cde^	19.24 ^cde^
		Ma	4.19 ^ef^	3.52 ^bc^	0.033 ^cd^	0.032 ^c^	96.00 ^abc^	20.66 ^dc^
		Cs-Ma	4.54 ^cd^	3.79 ^b^	0.042 ^bc^	0.032 ^c^	97.33 ^ab^	21.16 ^cd^
	Mehregan	Ck	3.71^fgh^	1.99 ^gh^	0.021 ^ef^	0.021 ^fgh^	89.33^de^	17.33 ^ef^
		Cs	3.37 ^hi^	2.04 ^gh^	0.024 ^def^	0.023 ^fg^	89.00^de^	18.00 ^de^
		Ma	3.75 ^fgh^	2.15 ^fgh^	0.029 ^de^	0.025 ^efg^	92.33 ^cde^	18.83 ^de^
		Cs-Ma	4.65 ^ab^	2.52 ^efg^	0.032 ^cd^	0.028 ^def^	94.66 ^cd^	19.14 ^cde^
−4 MPa	MV Nádor	Ck	2.31^ijk^	2.50 ^efg^	0.018 ^fg^	0.016 ^ghi^	78.66 ^gh^	14.83 ^efg^
		Cs	2.56 ^ij^	2.42 ^fg^	0.020 ^ef^	0.014 ^hij^	84.00 ^fg^	15.33 ^efg^
		Ma	2.69 ^hij^	2.67 ^def^	0.021 ^ef^	0.020 ^fgh^	88.66 ^de^	16.16 ^ef^
		Cs-Ma	3.31^hi^	2.96 ^cd^	0.024 ^def^	0.022 ^fg^	89.33^de^	18.33 ^de^
	Mehregan	Ck	2.13 ^k^	1.52 ^ij^	0.015 ^gh^	0.011 ^ij^	72.00 ^ij^	9.83 ^i^
		Cs	2.36 ^ijk^	1.54 ^ij^	0.017 ^fgh^	0.013 ^hij^	73.33 ^hij^	11.33 ^gh^
		Ma	2.59 ^ij^	1.61 ^hij^	0.019 ^fg^	0.017 ^ghi^	74.65 ^hi^	12.16 ^gh^
		Cs-Ma	2.75 ^hij^	2.41 ^fg^	0.023 ^def^	0.019 ^fgh^	77.33 ^ghi^	14.50 ^efg^

RL = radicle length, CL = coleoptile length, RW = radicle weight, CW = coleoptile weight, GP = germination percentage, GR = germination rate, control (Ck), chitosan (Cs), microalgae strain MACC-612 (Ma) and chitosan–microalgae (Cs-Ma). In each column, values followed by the same letter(s) do not have a significant difference at α = 0.05. Distilled water was used as a control.

**Table 3:** Susceptibility and tolerance indices for germination characteristics of Mehregan and MV Nádor under normal conditions and drought stress (−4 MPa).

Indices	Cultivars	Treatments	RL (cm)	CL (cm)	RW (g)	CW (g)	GP (%)	GR
		Ck	1.39 ^cd^	1.2 ^bc^	0.007 ^k^	0.013 ^j^	20.00 ^c^	10.4 ^bc^
	MV Nádor	Cs	1.16 ^de^	0.65 ^de^	0.007 ^k^	0.015 ^j^	14.66 ^d^	9.8 ^c^
		Ma	1.68 ^c^	1.07 ^c^	0.025 ^ij^	0.020 ^i^	10.00 ^e^	10.3 ^bc^
TOL		Cs-Ma	2.08 ^b^	1.61 ^b^	0.029 ^i^	0.020 ^i^	10.67 ^e^	10.8 ^b^
		Ck	2.36 ^a^	1.59 ^b^	0.01^jk^	0.020 ^i^	25.33 ^a^	12.3 ^a^
	Mehregan	Cs	1.29 ^d^	1.6 ^b^	0.008 ^k^	0.017 ^ij^	22.67 ^b^	11.0 ^ab^
		Ma	1.44 ^cd^	1.92 ^a^	0.015 ^j^	0.018 ^ij^	22.68 ^b^	12.7 ^a^
		Cs-Ma	1.84 ^bc^	1.3 ^bc^	0.016 ^j^	0.017 ^ij^	21.33 ^bc^	10.8 ^b^
		Ck	0.475 ^gh^	0.726 ^d^	0.413 ^ef^	0.516 ^ef^	0.805 ^fg^	0.595 ^ef^
	MV Nádor	Cs	0.529 ^g^	0.583 ^de^	0.496 ^de^	0.451 ^fg^	0.860 ^fg^	0.612 ^de^
		Ma	0.653 ^ef^	0.784 ^d^	0.887 ^b^	0.889 ^b^	0.908 ^f^	0.682 ^de^
STI		Cs-Ma	0.991^e^	1.061 ^c^	1.168 ^a^	1.027 ^a^	0.927 ^f^	0.851^d^
		Ck	0.531 ^g^	0.371 ^g^	0.344 ^fg^	0.379 ^gh^	0.727 ^h^	0.346 ^k^
	Mehregan	Cs	0.478 ^gh^	0.379 ^g^	0.390 ^f^	0.433 ^fg^	0.731 ^h^	0.403 ^hi^
		Ma	0.580 ^fg^	0.446 ^fg^	0.593 ^c^	0.661^d^	0.754 ^gh^	0.481 ^fg^
		Cs-Ma	0.701^ef^	0.702 ^d^	0.824 ^bc^	0.760 ^c^	0.792 ^gh^	0.584 ^ef^
		Ck	0.376 ^hi^	0.324 ^hi^	0.280 ^gh^	0.448 ^fg^	0.20 ^j^	0.412 ^h^
	MV Nádor	Cs	0.312 ^j^	0.212 ^j^	0.259 ^gh^	0.517 ^ef^	0.15 ^jk^	0.389 ^ij^
		Ma	0.384 ^hi^	0.286 ^ij^	0.543 ^cd^	0.500 ^f^	0.10 ^k^	0.390 ^ij^
SI		Cs-Ma	0.386 ^hi^	0.352 ^gh^	0.547 ^cd^	0.476 ^fg^	0.11 ^k^	0.371 ^jk^
		Ck	0.526 ^g^	0.511 ^ef^	0.400 ^f^	0.645 ^d^	0.26 ^i^	0.555 ^ef^
	Mehregan	Cs	0.353 ^ij^	0.510 ^ef^	0.320 ^fg^	0.567 ^de^	0.24 ^ij^	0.493 ^fg^
		Ma	0.357 ^ij^	0.544 ^e^	0.441 ^ef^	0.514 ^ef^	0.23 ^ij^	0.510 ^f^
		Cs-Ma	0.401^h^	0.350 ^gh^	0.410 ^f^	0.472 ^fg^	0.22 ^ij^	0.427 ^gh^

RL = radicle length, CL = coleoptile length, RW = radicle weight, CW = coleoptile weight, GP = germination percentage, GR = germination rate, control (Ck), chitosan (Cs), microalgae strain MACC-612 (Ma) and chitosan–microalgae (Cs-Ma). Tolerance index (TOL), stress tolerance index (STI) and stress intensity (SI). In each column, values followed by the same letter(s) do not have a significant difference at α = 0.05.

**Table 4:** Genes and primers used for RT-qPCR analyses in the present study.

Gene	Forward Primer (5′-3′)	Reverse Primer (5′-3′)
*TaActin*	GTGTACCCTCAGAGGAATAAGG	GTACCACACAATGTCGCTTAGG
*TaPAL*	CCATCACCAAGCTGCTCAAC	ATAAGGCCGGCAATGTAGG
*TaSAMDC*	ACAGCCTTCTCCACACAAGA	TCCAGACCAGTCATGCACA
*TaPXPAO*	GCTCATAAATCAGCCCAATTCCA	TTCGCCATTTGTTGAGCTCT
*TaHSP70*	GTACCACACAATGTCGCTTAGG	TCCTGGTTCAAAGCCTCCAT
*TaSPDS*	AGGTATTCAAGGGTGGCGTG	TGGGTTCACAGGAGTCAGGA

## Data Availability

All the data in the present study are included in this manuscript.

## Abbreviations

Chitosan (Cs); chitosan–microalgae (Cs-Ma); microalgae (Ma); polyethylene glycol (PEG), peroxisomal polyamine oxidase (PxPAO); S-adenosylmethionine decarboxylase (SAMDC); spermidine synthase (SPDS); phenylpropanoid metabolism (PAL); protein protection (HSP70); tolerance index (TOL); stress tolerance index (STI); stress intensity (SI).

## References

[1] Daryanto S., Wang L., Jacinthe P.-A. (2016). Global synthesis of drought effects on maize and wheat production. PLoS ONE.

[2] Bohra A., Choudhary M., Bennett D., Joshi R., Mir R.R., Varshney R.K. (2024). Drought-tolerant wheat for enhancing global food security. Funct. Integr. Genom..

[3] Sallam A., Alqudah A.M., Dawood M.F., Baenziger P.S., Börner A. (2019). Drought stress tolerance in wheat and barley: Advances in physiology, breeding and genetics research. Int. J. Mol. Sci..

[4] Ahmed K., Shabbir G., Ahmed M. (2025). Exploring drought tolerance for germination traits of diverse wheat genotypes at seedling stage: A multivariate analysis approach. BMC Plant Biol..

[5] Alcázar R., Altabella T., Marco F., Bortolotti C., Reymond M., Koncz C., Carrasco P., Tiburcio A.F. (2010). Polyamines: Molecules with regulatory functions in plant abiotic stress tolerance. Planta.

[6] Sharma A., Shahzad B., Rehman A., Bhardwaj R., Landi M., Zheng B. (2019). Response of phenylpropanoid pathway and the role of polyphenols in plants under abiotic stress. Molecules.

[7] Iriti M., Faoro F. (2009). Chitosan as a MAMP, searching for a PRR. Plant Signal. Behav..

[8] Hidangmayum A., Dwivedi P., Katiyar D., Hemantaranjan A. (2019). Application of chitosan on plant responses with special reference to abiotic stress. Physiol. Mol. Biol. Plants.

[9] Malerba M., Cerana R. (2019). Recent applications of chitin-and chitosan-based polymers in plants. Polymers.

[10] Shinde N.A., Kawar P.G., Dalvi S.G. (2024). Chitosan-based nanoconjugates: A promising solution for enhancing crops drought-stress resilience and sustainable yield in the face of climate change. Plant Nano Biol..

[11] Behboudi F., Tahmasebi-Sarvestani Z., Kassaee M.Z., Modarres-Sanavy S.A.M., Sorooshzadeh A., Mokhtassi-Bidgoli A. (2019). Evaluation of chitosan nanoparticles effects with two application methods on wheat under drought stress. J. Plant Nutr..

[12] Das D., Bisht K., Chauhan A., Gautam S., Jaiswal J.P., Salvi P., Lohani P. (2023). Morpho-physiological and Biochemical responses in wheat foliar sprayed with zinc-chitosan-salicylic acid nanoparticles during drought stress. Plant Nano Biol..

[13] Parmar P., Kumar R., Neha Y., Srivatsan V. (2023). Microalgae as next generation plant growth additives: Functions, applications, challenges and circular bioeconomy based solutions. Front. Plant Sci..

[14] Vangenechten B., De Coninck B., Ceusters J. (2025). How to improve the potential of microalgal biostimulants for abiotic stress mitigation in plants?. Front. Plant Sci..

[15] Kusvuran S. (2021). Microalgae (*Chlorella vulgaris Beijerinck*) alleviates drought stress of broccoli plants by improving nutrient uptake, secondary metabolites, and antioxidative defense system. Hortic. Plant J..

[16] Munaro D., Mazo C.H., Bauer C.M., da Silva Gomes L., Teodoro E.B., Mazzarino L., da Rocha Veleirinho M.B., e Silva S.M., Maraschin M. (2024). A novel biostimulant from chitosan nanoparticles and microalgae-based protein hydrolysate: Improving crop performance in tomato. Sci. Hortic..

[17] El Amerany F., Naimi M., Rhazi M. (2025). Biostimulant-driven growth enhancement and stress resistance in tomato: The combined impact of alginate, chitosan, and salicylic acid. J. Biotechnol..

[18] Pál M., Tajti J., Szalai G., Peeva V., Végh B., Janda T. (2018). Interaction of polyamines, abscisic acid and proline under osmotic stress in the leaves of wheat plants. Sci. Rep..

[19] Khan Z., Shahwar D. (2020). Role of heat shock proteins (HSPs) and heat stress tolerance in crop plants. Sustainable Agriculture in the Era of Climate Change.

[20] Yadav P., Singh R.P., Hashem A., Abd_Allah E.F., Santoyo G., Kumar A., Gupta R.K. (2023). Enhancing biocrust development and plant growth through inoculation of desiccation-tolerant cyanobacteria in different textured soils. Microorganisms.

[21] Bewley J.D., Bradford K., Hilhorst H. (2012). Seeds: Physiology of Development, Germination and Dormancy.

[22] Farooq M., Wahid A., Kobayashi N., Fujita D., Basra S.M. (2009). Plant drought stress: Effects, mechanisms and management. Sustainable Agriculture.

[23] Fernandez G.C. (1992). Effective selection criteria for assessing plant stress tolerance. Adaptation of Food Crops to Temperature and Water Stress.

[24] Fischer R., Maurer R. (1978). Drought resistance in spring wheat cultivars. I. Grain yield responses. Aust. J. Agric. Res..

[25] Hadwiger L.A. (2013). Multiple effects of chitosan on plant systems: Solid science or hype. Plant Sci..

[26] Bulgari R., Franzoni G., Ferrante A. (2019). Biostimulants application in horticultural crops under abiotic stress conditions. Agronomy.

[27] Wang X., He M., Wang X., Liu S., Luo L., Zeng Q., Wu Y., Zeng Y., Yang Z., Sheng G. (2024). Emerging nanochitosan for sustainable agriculture. Int. J. Mol. Sci..

[28] Akdaşçi E., Duman H., Eker F., Bechelany M., Karav S. (2025). Chitosan and its nanoparticles: A multifaceted approach to antibacterial applications. Nanomaterials.

[29] Alcázar R., Bueno M., Tiburcio A.F. (2020). Polyamines: Small Amines with Large Effects on Plant Abiotic Stress Tolerance. Cells.

[30] Gondor O.K., Tajti J., Hamow K.Á., Majláth I., Szalai G., Janda T., Pál M. (2021). Polyamine Metabolism under Different Light Regimes in Wheat. Int. J. Mol. Sci..

[31] Moschou P., Wu J., Cona A., Tavladoraki P., Angelini R., Roubelakis-Angelakis K. (2012). The polyamines and their catabolic products are significant players in the turnover of nitrogenous molecules in plants. J. Exp. Bot..

[32] Pál M., Szalai G., Janda T. (2015). Speculation: Polyamines are important in abiotic stress signaling. Plant Sci..

[33] Gholizadeh F., Janda T., Gondor O.K., Pál M., Szalai G., Sadeghi A., Turkoglu A. (2022). Improvement of Drought Tolerance by Exogenous Spermidine in Germinating Wheat (*Triticum aestivum* L.) Plants Is Accompanied with Changes in Metabolite Composition. Int. J. Mol. Sci..

[34] Dixon R.A., Paiva N.L. (1995). Stress-induced phenylpropanoid metabolism. Plant Cell.

[35] Wang K., Zhang X., Goatley M., Ervin E. (2014). Heat shock proteins in relation to heat stress tolerance of creeping bentgrass at different N levels. PLoS ONE.

[36] Michel B.E., Kaufmann M.R. (1973). The osmotic potential of polyethylene glycol 6000. Plant Physiol..

[37] Tamiya H. (1957). Mass culture of algae. Annu. Rev. Plant Physiol..

[38] Ördög V. (1982). Apparatus for laboratory algal bioassay. Int. Rev. Gesamten Hydrobiol..

[39] Mutum L., Janda T., Darkó É., Szalai G., Hamow K.Á., Molnár Z. (2023). Outcome of microalgae biomass application on seed germination and hormonal activity in winter wheat leaves. Agronomy.

[40] Rosielle A., Hamblin J. (1981). Theoretical aspects of selection for yield in stress and non-stress environment 1. Crop Sci..

[41] Gholizadeh F., Gohari G., Pál M., Szalai G., Khan I., Janda T. (2025). Enhancing wheat resilience to salt stress through an integrative nanotechnology approach with chitosan proline and chitosan glycine. Sci. Rep..

[42] Livak K.J., Schmittgen T.D. (2001). Analysis of relative gene expression data using real-time quantitative PCR and the 2^−ΔΔCT^ method. Methods.

[43] Pál M., Hamow K.Á., Rahman A., Majláth I., Tajti J., Gondor O.K., Ahres M., Gholizadeh F., Szalai G., Janda T. (2022). Light Spectral Composition Modifies Polyamine Metabolism in Young Wheat Plants. Int. J. Mol. Sci..

[44] Amirbakhtiar N., Ismaili A., Ghaffari M.-R., Mirdar Mansuri R., Sanjari S., Shobbar Z.-S. (2021). Transcriptome analysis of bread wheat leaves in response to salt stress. PLoS ONE.

[45] Majláth I., Benczúr K., Rahman A., Janda T., Likó I., Kádas J., Pál M. (2025). Putrescine treatment has a higher effect on 5mC DNA methylation profile of wheat leaves under white than under blue light conditions. Sci. Rep..

[46] Steel R.G.D., Torrie J.H. (1981). Principles and Procedures of Statistics, a Biometrical Approach.

[47] SAS Institute (2018). SAS 9.4 Language Reference: Concepts.

